#  Corrigendum

**DOI:** 10.1002/ccr3.6166

**Published:** 2022-09-14

In the article entitled “Recurrent pericarditis as an extra‐intestinal manifestation of ulcerative colitis in a 14‐year‐old girl,” which was previously published in Volume 6 Issue 8 of Clinical Case Reports, the given statement was published incorrectly. “Sulfasalazine was initiated as maintenance therapy of UC. Colchicine (0.5 g bidaily, orally) was added to the treatment to prevent the recurrence of the pericarditis.”

It has been corrected as follows: “Sulfasalazine was initiated as maintenance therapy of UC. Colchicine (0.5 mg bidaily, orally) was added to the treatment to prevent the recurrence of the pericarditis.”
